# Brain-age is associated with progression to dementia in memory clinic patients

**DOI:** 10.1016/j.nicl.2022.103175

**Published:** 2022-08-30

**Authors:** Francesca Biondo, Amelia Jewell, Megan Pritchard, Dag Aarsland, Claire J. Steves, Christoph Mueller, James H. Cole

**Affiliations:** a Department of Neuroimaging, Institute of Psychiatry, Psychology and Neuroscience, King’s College London, SE5 8AF, UK; b South London and Maudsley NHS Foundation Trust, UK; c Centre for Medical Image Computing, Department of Computer Science, University College London, WC1V 6LJ, UK; d Department of Old Age Psychiatry, Institute of Psychiatry, Psychology and Neuroscience, King’s College London, SE5 8AF, UK; e Centre for Age-Related Research, Stavanger University Hospital, Stavanger, Norway; f Department of Ageing and Health, Guy’s and St Thomas’ NHS Foundation Trust, SE1 7EH, UK; g Department of Twin Research and Genetic Epidemiology, King’s College London, SE1 7EH, UK; h Dementia Research Centre, Institute of Neurology, University College London, WC1N 3AR, UK

**Keywords:** Brain-age, Machine learning, Dementia, Ageing, Electronic health records, Ecological validity, brain-PAD, Brain Predicted Age Difference, futureDD, future Dementia Diagnosis patient group, noDD, no Dementia Diagnosis patient group, MMSE, Mini-Mental State Examination

## Abstract

**Background:**

Biomarkers for the early detection of dementia risk hold promise for better disease monitoring and targeted interventions. However, most biomarker studies, particularly in neuroimaging, have analysed artificially ‘clean’ research groups, free from comorbidities, erroneous referrals, contraindications and from a narrow sociodemographic pool. Such biases mean that neuroimaging samples are often unrepresentative of the target population for dementia risk (e.g., people referred to a memory clinic), limiting the generalisation of these studies to real-world clinical settings. To facilitate better translation from research to the clinic, datasets that are more representative of dementia patient groups are warranted.

**Methods:**

We analysed T1-weighted MRI scans from a real-world setting of patients referred to UK memory clinic services (*n* = 1140; 60.2 % female and mean [*SD*] age of 70.0[10.8] years) to derive ‘brain-age’. Brain-age is an index of age-related brain health based on quantitative analysis of structural neuroimaging, largely reflecting brain atrophy. Brain-predicted age difference (brain-PAD) was calculated as brain-age minus chronological age. We determined which patients went on to develop dementia between three months and 7.8 years after neuroimaging assessment (*n* = 476) using linkage to electronic health records.

**Results:**

Survival analysis, using Cox regression, indicated a 3 % increased risk of dementia per brain-PAD year (hazard ratio [95 % *CI*] = 1.03 [1.02,1.04], *p* < 0.0001), adjusted for baseline age, age^2^, sex, Mini Mental State Examination (MMSE) score and normalised brain volume. In sensitivity analyses, brain-PAD remained significant when time-to-dementia was at least 3 years (hazard ratio [95 % CI] = 1.06 [1.02, 1.09], *p* = 0.0006), or when baseline MMSE score ≥ 27 (hazard ratio [95 % CI] = 1.03 [1.01, 1.05], *p* = 0.0006).

**Conclusions:**

Memory clinic patients with older‐appearing brains are more likely to receive a subsequent dementia diagnosis. Potentially, brain-age could aid decision-making during initial memory clinic assessment to improve early detection of dementia. Even when neuroimaging assessment was more than 3 years prior to diagnosis and when cognitive functioning was not clearly impaired, brain-age still proved informative. These real-world results support the use of quantitative neuroimaging biomarkers like brain-age in memory clinics.

## Highlights

• Brain-age is an index of the brain’s ‘biological’ age based on T1-weighted MRI data.
• Memory clinic patients with older-appearing brains have higher risk of dementia.
• Results are independent of medical history, age, sex, MMSE score and brain volumes.
• Brain-age has the potential to aid early detection of dementia in patients.

## 1 Introduction

The growing global burden of dementia motivates research to improve the early identification of people at highest risk of developing the disease. Early dementia-risk identification has important implications for future care planning, the timing of possible interventions and for stratified clinical trial enrolment. Investigations into markers of dementia risk abound and include cognitive and behavioural markers (Belleville et al., 2017, Montero-Odasso et al., 2017) as well as biomarkers relating to fluid protein levels (e.g., blood or CSF measures of amyloid, tau, neurofilament light) or brain structure, function or metabolism (Humpel, 2011, Jack et al., 2018). However, despite these promising findings, translation into clinical practice has been limited (Gamo et al., 2017, Cole, 2018). One potential explanation for this is a lack of ecological validity; do findings generalise from laboratory to natural (e.g., clinical) contexts? (Burgess et al., 2006, Kvavilashvili and Ellis, 2004). Poor ecological validity is often due to study samples not being representative of the real-world population of interest. Unrepresentative samples can be caused by selection bias, where participants with certain characteristics are more or less likely to be included in the study than others (Simundić, 2013, Prince, 2012). For example, recruiting patients from a dementia clinic whilst selecting controls from a primary care clinic may lead to potentially erroneous conclusions, such as arthritis and cataracts being more common in controls (Broe et al., 1990). Another example is the large prospective cohort study, UK Biobank, which displays a ‘healthy volunteer’ bias whereby the included participants were found to be more health-conscious compared with the general population (Fry et al., 2017). The demographics of research volunteers may also not be representative of the target population, in terms of age, socio-economic status and ethnicity. Typically, dementia-risk studies exclude participants with current or past comorbidities to reduce heterogeneity (Dickerson et al., 2011, Li et al., 2012, Vemuri et al., 2010). However, comorbidities are common in people at risk of dementia (Bunn et al., 2014, Livingston et al., 2020, Browne et al., 2017) and could play a role in disease progression, meaning that clinicians need to account for them in practice. Properly representative research studies should strive to do the same.

Neuroimaging is a strong candidate for identifying early dementia risk, particularly MRI, as it is routinely collected in clinical contexts (Rathore et al., 2017). Previous work has been consistent in reporting brain atrophy (Vemuri et al., 2010, Plant et al., 2010), cortical thinning (Dickerson et al., 2011, Li et al., 2012), microstructural abnormalities including white-matter hyperintensities (Prins and Scheltens, 2015, Zeestraten et al., 2017) and differences in functional connectivity (Hohenfeld et al., 2018) as associated to future dementia. One promising approach to investigate health outcomes in neurodegenerative diseases is the brain-age paradigm. Brain-age is an index of the brain’s biological age, with previous studies supporting the idea that ‘older’-appearing brains are indicative of a greater risk of age-associated brain diseases and poor health outcomes, including mortality (Cole and Franke, 2017, Kaufmann et al., 2019, Han et al., 2020, Franke and Gaser, 2019, Cole et al., 2018, Cole et al., 2019, Cole et al., 2020). Brain-age has also been associated with subsequent dementia in observational research cohorts (Gaser et al., 2013, Wang et al., 2019). When compared with other Alzheimer’s disease biomarkers such as CSF-based amyloid and tau markers, or PET markers, brain-age provided an independent contribution in identifying people who convert from mild cognitive impairment to Alzheimer’s disease (Popescu et al., 2020).

A key limitation of these initial brain-age studies is that the samples they use are unrepresentative of the general population at-risk for dementia. Research participants are likely to be more highly educated, have a higher IQ, be less ethnically diverse and have fewer comorbidities than the general population (Petersen et al., 2010). Even in research studies that have aimed to obtain a representative sample (Ikram et al., 2011), the aforementioned healthy-volunteer bias is challenging to overcome and factors such as the threshold for contraindications for undergoing MRI will differ between research and clinical settings.

Here, we sought to improve ecological validity using a large real-world dataset of patients referred to memory clinics for MRI assessment. This dataset is naturally representative as inclusion in the study was based on clinical need, not research-related criteria. The opportunity to investigate those memory clinic patients referred for neuroimaging assessment is particularly relevant to clinical translation; an MRI scan is typically requested in cases of diagnostic ambiguity and thus represents a key stage on the patient pathway. This retrospective study analysed structural MRI scans of memory clinic patients whose future clinical status (i.e., presence or absence of dementia) was determined via linkage to electronic health records and we hypothesised that brain-age would be significantly associated to a subsequent dementia diagnosis.

## 2 Material and methods

### 2.1 Patients

This study analysed data from 1140 memory clinic patients who were referred for neuroimaging assessment, as part of routine care at the South London and the Maudsley National Health Service Foundation Trust. The South London and the Maudsley National Health Service Foundation Trust is one of the largest secondary mental healthcare providers in Europe, serving over 1.36 million residents from predominantly-four London boroughs (Croydon, Lambeth, Lewisham and Southwark) (Stewart et al., 2009, Perera et al., 2016). The earliest scan date in our sample is 28/01/2011. Access to these data was obtained as part of two ongoing studies (BRCMEM and BRCDEM, Biomedical Research Centre Memory and Dementia, studies).

Patients’ demographic, including self-reported ethnicity, and clinical data were available from de-identified electronic health records which were accessed via the Clinical Record Interactive Search based at the Maudsley NIHR Biomedical Research Centre (Stewart et al., 2009, Perera et al., 2016, NIHR BRC, 2021). The Clinical Record Interactive Search is a clinical database with a robust data governance framework with ethical approval for secondary data analysis (Oxford REC C reference 18/SC/0372). The Clinical Record Interactive Search provided linkage between electronic health records and the memory clinic neuroimaging data, as well as linkage to two other datasets, the Hospital Episode Statistics (HES) and the Office of National Statistics (ONS) Mortality database. HES is a national dataset that contains data of outpatient appointments and hospital admissions at NHS hospitals in England (Academy of Medical Royal Colleges, 2011). The ONS Mortality database contains the date and cause of death for all deaths registered in England and Wales (Primary care mortality database, 2019). HES and the ONS Mortality datasets were used to supplement the electronic health records with diagnostic and mortality data.

**Fig. 1:**
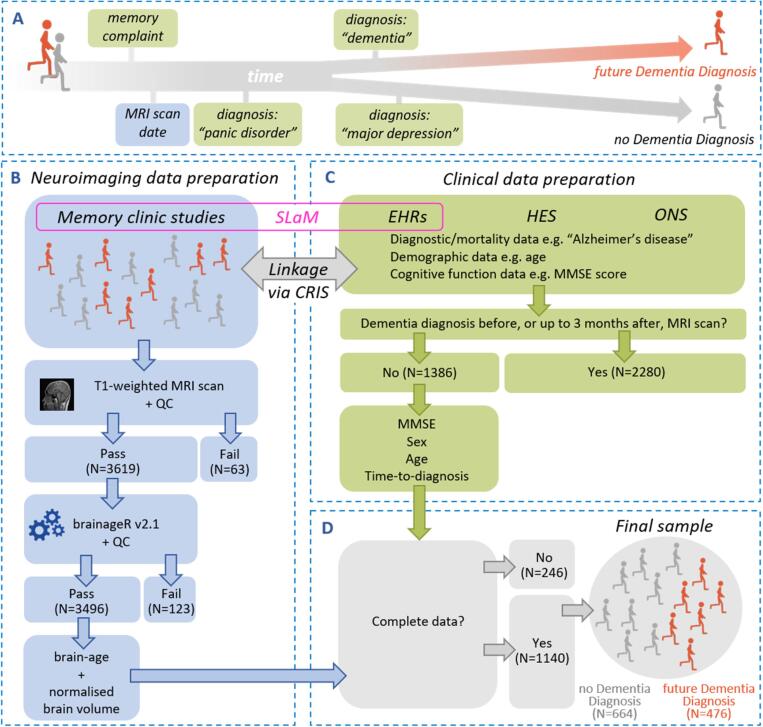
**Data pre-processing pipeline.** (**A**) Memory clinic patients were classified as: “future Dementia Diagnosis” (orange) or “no Dementia Diagnosis” (grey). (**B**) Neuroimaging data preparation: brainageR was applied to T1-weighted MRI scans to obtain a brain-age estimate and normalised brain volume for each patient. Quality Control (QC) aimed to remove cases with image artefacts and occurred at two stages, before and after segmentation. (**C**) Clinical data preparation: clinical data was retrieved across three databases, South London and Maudsley Hospital Electronic Health Record (SLaM EHRs), Hospital Episodes Statistics (HES) and Office of National Statistics (ONS) which were accessed and linked to the neuroimaging data via the Clinical Record Interactive Search (CRIS). These databases provided diagnostic, demographic, cognitive and mortality data and facilitated labelling into “future Dementia Diagnosis” or “no Dementia Diagnosis”. Patients who were diagnosed with dementia before, or up to 3 months after the neuroimaging assessment, were excluded. (**D**) Merging the neuroimaging and clinical data: the final dataset (N = 1140) included only complete cases for the following variables: sex, age, brain-age, normalised brain volume, MMSE (Mini Mental State Examination) and scanner information. (For interpretation of the references to colour in this figure legend, the reader is referred to the web version of this article.)

Permission to access electronic health records, HES, the ONS Mortality database and the memory clinic neuroimaging dataset was granted via Clinical Record Interactive Search (reference 19–008) and accessed on 11/10/2019. Informed consent for research-use of neuroimaging data was obtained from the participants at time of assessment (BRCMEM and BRCDEM studies). Data preparation was carried out via two routes, clinical and neuroimaging (Fig. 1).This study was pre-registered (aspredicted.org ref.26262) before data access.

### 2.2 Clinical data preparation

The Clinical Record Interactive Search enabled linkage of neuroimaging data to electronic health records, identified an initial total of 3666 patients. Of these, 2472 were classified as “future Dementia Diagnosis” (*futureDD*) patients and 1194 as “no Dementia Diagnosis” (*noDD*) patients. Classification was achieved by extraction of diagnostic information and/or cause of death across electronic health records. A dementia diagnosis was operationalised as a positive search result for the following terms: ICD-10 codes F00-F03, G30-G32, “dementia”, “Alzheimer’s disease”, “Alzheimer” and “Lewy”. The search was carried out in both structured and unstructured fields of the electronic health records. Unstructured fields included free-text clinical notes and the search was achieved via natural language processing applications developed and validated within the Clinical Record Interactive Search at the Maudsley NIHR Biomedical Research Centre (Perera et al., 2016, NIHR BRC, 2021). A negative search result classified the patient as *noDD*.

For the *futureDD* patients, the diagnosis time was the date of the first instance of a dementia diagnosis after the neuroimaging assessment. For the *noDD* patients, the diagnosis time was the last instance of a diagnostic clinical entry after the neuroimaging assessment. A dementia diagnosis was detected before the neuroimaging assessment in 745 dementia patients (suggesting the diagnosis had been made based on clinical assessment after referral to the MRI unit, but before the actual scan); these were excluded. Seventy-two *noDD* cases were excluded because their most recent non-dementia diagnosis preceded the neuroimaging assessment.

No exclusion were based on medical history (except dementia, as previously described). This deviates from our pre-registered statement because, in retrospect, we opted to better capture the heterogeneity of memory clinic patients as this would increase ecological validity.

As the goal of the study was to assess the value of brain-age for early identification of future dementia, we opted to exclude patients for whom the MRI scan was used diagnostically (as opposed to prognostically). To focus on prognostic identification of future dementia, we set a minimum threshold of 3 months between neuroimaging assessment and diagnosis, based on South London and the Maudsley National Health Service Foundation Trust clinical guidelines of the maximum wait for a diagnosis. This reduced the sample from 2848 to 1386 patients.

In addition to diagnostic information, the following variables ascertained at, or closest to, the neuroimaging assessment were obtained from the electronic health records: age, sex, Mini-Mental State Examination (MMSE) and Addenbrookes’ Cognitive Examination. The MMSE and Addenbrookes’ Cognitive Examination are brief cognitive functioning tests commonly used in the clinic and helpful at detecting dementia (Burns et al., 1975, Hodges and Larner, 2016). The scores range from 0 to 30 and 0–100, respectively, with low scores indicating poor cognition. Natural language processing applications (validated within the Clinical Record Interactive Search at the Maudsley NIHR Biomedical Research Centre) (Perera et al., 2016, NIHR BRC, 2021) were used to extract MMSE and Addenbrookes’ Cognitive Examination scores from both structured and unstructured electronic health records fields.

### 2.3 Neuroimaging data preparation

In total, 3682 T1-weighted MRI scans were accessed. These were acquired at 1.5 T using GE scanners (General Electric, WI, US), with generally similar but not identical acquisition parameters (see Appendix A). Visual quality control was conducted to detect image artefacts; 63 scans were excluded due to poor quality such as motion artefacts and field inhomogeneities.

Brain age was calculated using brainageR (v2.1), an open-access software for generating brain-predicted age from raw T1-weighted MRI scans (https://github.com/james-cole/brainageR) (Cole et al., 2018). BrainageR involves two main stages, pre-processing and prediction. In the pre-processing stage, images were segmented and normalised via SPM12 software (https://www.fil.ion.ucl.ac.uk/spm/software/spm12/). For quality control, the FSL *slicesdir* function was used to generate two-dimensional slices the segmentation and normalisation outputs; 123 images were excluded due to gross segmentation errors. Normalised images were loaded into *R* (R Core Team, 2019) and converted to vectors. Grey matter (GM), white matter (WM) and cerebrospinal fluid (CSF) vectors were masked using a 0.3 threshold from the mean image template based on the brainageR model training dataset and then combined.

In the prediction stage, the brainageR model was applied to the vectorised and masked study images to estimate a brain-age score for each. BrainageR had been previously trained to predict age from normalised brain volumetric maps of *n* = 3377 healthy individuals from seven publicly available datasets using a Gaussian Processes Regression (see Appendix B for a list of training and testing datasets**)**. Using principal component analysis, the top principal components capturing 80 % of the variance in brain volumes were retained. The resulting rotation matrix for 435 principal components was then applied to the new imaging data to predict age. Model performance (bivariate correlation between chronological age and brain-predicted age, *r*; mean absolute error, MAE) for internal and external validation was: *n* = 857, *r* = 0.973, MAE = 3.933 years and *n* = 611, *r* = 0.947, MAE = 4.90 years, respectively (https://github.com/james-cole/brainageR) (Cole et al., 2018).

For each image, the final output of brainageR was a brain-predicted age value with 95 % confidence intervals (*CI*). Brain-predicted age difference (brain-PAD) was calculated by subtracting chronological age from brain-predicted age. Volumetric measures of GM, WM and CSF were generated by SPM Segment. Normalised brain volume was calculated as the sum of GM and WM volumes, divided by the sum of GM, WM, and CSF volumes.

Neuroimaging data were subsequently merged with the clinical data. Out of 1386 cases, 81 were removed because of a missing brain-age score or a different MRI scanner and 165 were removed because of missing MMSE scores. In summary, the initial sample of 3666 cases was finally reduced to 1140 patients after the various pre-processing steps described above. The final sample (*n* = 1140) consisted of 664 *futureDD* and 476 *noDD* patients.

### 2.4 Statistical analyses

To examine whether baseline brain-PAD was associated with subsequent dementia diagnosis (after at least 3 months), we ran a survival analysis using Cox proportional hazards regression. Cox regression allows for the modelling of the time-to-event (time-to-dementia), also referred to as survival or event time. For the *noDD* patients, time-to-dementia was right-censored using the data of the most recent diagnostic clinical entry. The explanatory variables were brain-PAD alongside covariates of sex, age, age^2^, MMSE score and normalised brain volume. Age and age^2^ were included to address the bias due to the correlation between chronological age and brain-PAD (de Lange and Cole, 2020). The problem of multicollinearity between age and age^2^ was minimized by orthogonalizing these variables using the ‘poly’ function in *R* (R Core Team, 2019).

Next, we ran three sensitivity analyses by sub-setting the sample data and re-running the Cox regression analysis. Firstly, we examined whether brain-PAD was associated to the risk of dementia over a longer period, using a minimum duration of 3 years between neuroimaging assessment and diagnosis. A threshold of three years was chosen to optimise maximum duration whilst retaining a sufficiently large and balanced sample. Secondly, we tested whether brain-PAD was associated to the risk of dementia in patients appearing to be largely cognitively unimpaired, by excluding those with baseline MMSE scores below 27, a value previously proposed as a conservative cut-off for dementia (O'Bryant et al., 2008). In addition, considering that a few patients (*n* = 101) were younger than expected for a memory clinic patient group (e.g., age 27), we ran a third sensitivity analysis with the broad aim to exclude these younger cases by including only patients 55 years or older. Finally, we tested for potential issues of multicollinearity in our main analysis by examining intercorrelations and variance inflation factors (VIF) of the predictors. VIF indicates how much the standard error of a predictor variable would change due to the inclusion of a potentially collinear covariate.

The data in this study are not available due to restrictions pertaining to information that could compromise the privacy of the patients. However, the analyses code with extended derived data can be accessed here: https://github.com/biondof/BARCODE.

## 3 Results

Baseline characteristics of the final sample (*n* = 1140) are described in Table 1 (see Appendix C for histograms**)**. At the time of neuroimaging assessment, 60.18 % were female, the mean age was 69.99 (*SD* 10.80) years, the mean MMSE score was 23.01 (*SD* 6.90), the mean Addenbrookes’ Cognitive Examination score was 72.30 (*SD* 15.63) and the mean normalised brain volume was 0.7239 (*SD* 0.0591) litres. Ethnicity data is depicted in Appendix D. Compared with the *noDD* group, the *futureDD* group had more females, was older, had lower MMSE and Addenbrookes’ Cognitive Examination scores and had smaller brain volumes. The median time-to-dementia diagnosis was 0.81 years (interquartile range 0.41–1.92).

**Table 1:** Baseline sample characteristics.

**Group**	**All**	**no Dementia Diagnosis**	**future Dementia Diagnosis**	**group comparison**^4^ **(*p*-value)**
**N**	1140	664	476	
**Female %**	60.18	57.68	63.66	0.0422
**Age**				
Complete cases %	100.00	100.00	100.00	
Mean (*SD*)	69.99 (10.80)	66.95 (11.12)	74.24 (8.74)	<0.0001
Median	71.00	68.00	75.00	
Min-Max	27.00–95.00	27.00–95.00	42.00–95.00	
**MMSE**^1^				
Complete cases %	100.00	100.00	100.00	
Mean (*SD*)	23.01 (6.90)	23.88 (6.59)	21.79 (7.15)	<0.0001
Median	26.00	26.00	24.00	
Min-Max	0.00–30.00	1.00–30.00	0.00–30.00	
**Time-to-Dementia**^2^				
Complete cases %	100.00	100.00	100.00	
Mean (SD)	1.86 (1.61)	2.18 (1.66)	1.41 (1.42)	
Median	1.29	1.70	0.81	
Min-Max	0.25–7.81	0.25–7.81	0.25–7.49	
**ACE**^3^				
Complete cases %	35.09	39.31	29.20	
Mean (*SD*)	72.30 (15.63)	74.81 (14.82)	67.58 (16.05)	<0.0001
Median	75.00	78.00	70.00	
Min-Max	22.00–99.00	26.00–99.00	22.00–96.00	
**Normalised brain volume**				
Complete cases %	100.00	100.00	100.00	
Mean (*SD*)	0.7239 (0.0591)	0.7405 (0.0571)	0.7009 (0.0538)	<0.0001
Median	0.7261	0.7453	0.7073	
Min-Max	0.4728–0.8800	0.4738–0.8800	0.4728–0.8633	

1 MMSE = Mini-Mental State Examination score.

2 Time-to-Dementia = gap in time (years) between baseline and the first instance of a dementia diagnosis in the ‘future Dementia Diagnosis’ patients whilst, for the ‘no Dementia Diagnosis’ patients this is not time to a dementia diagnosis as by group definition they did not receive such diagnosis, instead this is right-censored using the most recent non-dementia diagnostic event.

3 ACE = Addenbrookes’ Cognitive Examination.

4 group comparison = ‘no Dementia Diagnosis’ patients vs ‘future Dementia Diagnosis’ patients.

**Fig. 2:**
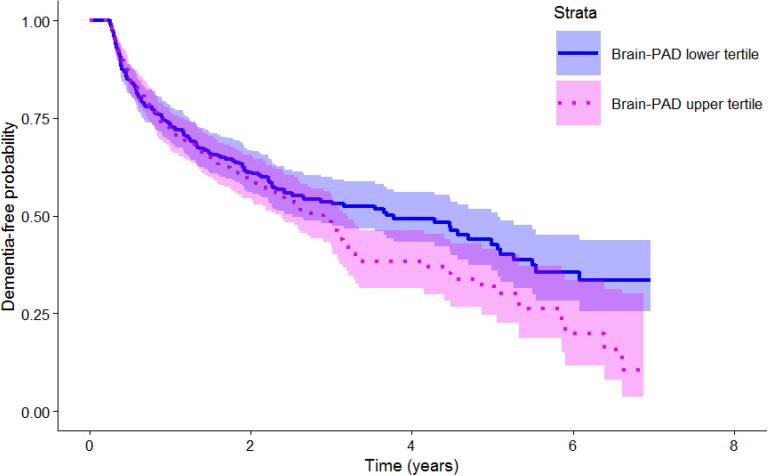
**Kaplan-Meier plot for brain-PAD**. This plot illustrates the proportion of patients who develop dementia based on a tertile split of brain-PAD score. At time 0 (time of the neuroimaging assessment, all patients are free of a dementia diagnosis. Over time, patients with higher brain-PAD scores (pink) are more likely to get a dementia diagnosis and more rapidly than the ones with lower brain-PAD scores (blue). (For interpretation of the references to colour in this figure legend, the reader is referred to the web version of this article.)

Associations between brain-PAD and time-to-dementia diagnosis (Cox regression) are presented in Table 2. A higher brain-PAD was significantly associated with time to a future dementia diagnosis: hazards ratio (*HR*) = 1.03 [*CI* = 1.02–1.04]. Age, sex, MMSE and normalised brain volume, but not age^2^, were also significantly associated with time-to-dementia (Table 2). These results indicated that while keeping the covariates constant, every + 1 year of brain-PAD was accompanied with a 3 % relative increased risk of a future dementia diagnosis (*p* < 0.0001) which considered time-to-dementia (Fig. 2).

**Table 2:** Association of brain-PAD and all covariates, with incident dementia assessed by Cox proportional hazards models, in the total study sample and in subsamples based on sensitivity analyses.

		**Cox regression**			
	***n/N***	***HR***	**(95 % *CI*)**	***p*-value**	
**Main analysis**					
brain-PAD	476/1140	1.03	(1.02–1.04)	<0.0001	***
age^†^	476/1140	1.14 × 10^9^	(6.89 × 10^6^-1.90 × 10^11^)	<0.0001	***
age^2†^	476/1140	0.02	(0.00–1.60)	0.0808	
sex (M)	476/1140	0.74	(0.61–0.90)	0.0020	**
MMSE	476/1140	0.98	(0.97–0.99)	<0.0001	***
normalised brain volume	476/1140	4.40 × 10^-3^	(8.66 × 10^-4^-2.24 × 10^-2^)	<0.0001	***

**Sensitivity analysis I (**minimum 3 years for Time-to-Dementia)					
brain-PAD	66/249	1.06	(1.02–1.09)	0.0006	***
age^†^	66/249	4.71 × 10^6^	(2.23 × 10^3^-9.90 × 10^10^)	<0.0001	***
age^2†^	66/249	0.02	(0.00–6.80)	0.1951	
sex (M)	66/249	0.55	(0.32–0.95)	0.0327	*
MMSE	66/249	1.00	(0.97–1.04)	0.9015	
normalised brain volume	66/249	0.84	(0.01–127.17)	0.9468	

**Sensitivity analysis II** (MMSE score ≥ 27 at baseline)					
brain-PAD	146/471	1.03	(1.01–1.05)	0.0006	***
age^†^	146/471	1.22 × 10^7^	(2.47 × 10^4^-6.01 × 10^9^)	<0.0001	***
age^2†^	146/471	0.48	(0.00–61.00)	0.7649	
sex (M)	146/471	0.83	(0.59–1.16)	0.2674	
MMSE	146/471	0.86	(0.74–1.02)	0.0782	
normalised brain volume	146/471	5.17 × 10^-3^	(2.38 × 10^-4^-0.11)	0.0008	***

**Sensitivity analysis III** (age ≥ 55 years at baseline)					
brain-PAD	469/1039	1.03	(1.02–1.04)	<0.0001	***
age^†^	469/1039	3.78 × 10^5^	(1.02 × 10^4^-1.39 × 10^7^)	<0.0001	***
age^2†^	469/1039	0.44	(0.03–7.89)	0.5810	
sex (M)	469/1039	0.75	(0.62–0.91)	0.0030	**
MMSE	469/1039	0.98	(0.97–0.99)	0.0001	***
normalised brain volume	469/1039	5.50 × 10^-3^	(1.07 × 10^-3^-0.03)	<0.0001	***

*HR* = Hazards Ratio; *CI* = Confidence Intervals; *n* = number of ‘future Dementia Diagnosis’ patients; *N* = total number of patients; *** *p*-value < 0.001; ** *p*-value < 0.01; * *p*-value < 0.05.

All HR values are adjusted for covariates.

^†^To minimize potential collinearity between age and age^2^ we orthogonalized these variables using the R function ‘poly’. However, a caveat with orthogonalized terms is that these can lead to uninterpretable coefficients. Extended analyses outputs excluding polynomial terms can be accessed via the analyses code (see Methods section 2.4 for the web link).

Assumptions for the Cox regression model was met: the standardised residuals of the covariates were not correlated to event time indicating proportional hazards (*χ*^2^(6) = 11.5, *p* = 0.074). Intercorrelations between covariates were low, aside from age and normalised brain volume (r = −0.45) and the VIF values were <2 for all predictors (see Appendix E).

**Fig. 3:**
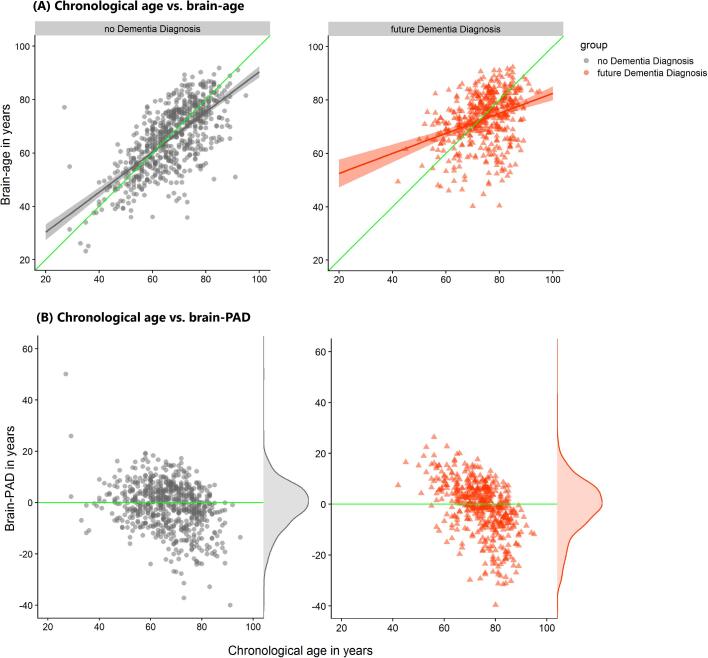
**Scatterplots of age, brain-age and brain-PAD**. The scatterplots show on the x-axis, chronological age and on the y-axis either brain-age (top panel A) or brain-PAD (bottom panel B) split by group, “no Dementia Diagnosis” (*noDD*; grey and on the left) and “future Dementia Diagnosis” (*futureDD*; orange and on the right). (**A**) Chronological age vs brain-age: The identity line (green) shows the ideal case when chronological age matches the brain-age estimate, y = x. Lines of best fit (orange, grey) within each group are both positive showing that brain-age estimates tend to be larger than chronological age for both groups of patients and in particular for the “future Dementia Diagnosis” group. (**B**) Chronological age vs brain-PAD: The density plots on the right of each scatterplot illustrate the distribution of brain-PAD scores. The green line is set at brain-PAD = 0 (i.e., brain age matches chronological age). (For interpretation of the references to colour in this figure legend, the reader is referred to the web version of this article.)

Congruent with our main results, the *noDD* group’s brain-age showed a relatively smaller deviation from their chronological age compared with the *futureDD* group, as illustrated in Fig. 3**A,** with the line of best fit closely aligned to the identity line in green (left) but less so for the *futureDD* group (right). Also, the deviation negatively correlated with age, likely driven by a regression-to-the-mean effect, hence we accounted for this in our analyses by covarying for age and age^2^ (de Lange and Cole, 2020). Critically, the brain-PAD scores were higher on average in the *futureDD* group, reflecting older-looking brains when compared to the *noDD* group (Fig. 3**B**).

Sensitivity analysis I, restricted to patients with a time-to-diagnosis minimum of 3 years (n = 249), resulted in *HR* = 1.06 [*CI* = 1.02–1.09], which was statistically significant. This indicated that for every + 1 year of brain-PAD there was a 6 % relative increased risk of a future dementia diagnosis, even when the neuroimaging assessment preceded diagnosis by at least 3 years. Sensitivity analysis II restricted to patients with baseline MMSE scores of above 26 (*n* = 471), resulted in *HR* = 1.03 (*CI* = 1.01–1.05) which was statistically significant. This indicated a 3 % increased risk of future dementia for every + 1 brain-PAD year, even when cognition was not clearly impaired. Sensitivity analysis III which included only patients with minimum 55 years of age, effectively reducing the sample by 101 cases, did not alter the main results. For sensitivity analyses results, see Table 2.

## 4 Discussion

In this study, we hypothesised that brain-age, a structural MRI-based biomarker, would be associated with subsequent dementia diagnoses in a real-world sample of memory clinic patients. We found that every additional year of brain-PAD incurred a 3 % relative increased risk of a future dementia diagnosis. Memory patient clinics with older-appearing brains at baseline (time of neuroimaging assessment), were more likely to receive a subsequent dementia diagnosis, independent of sex, baseline age, MMSE score and normalised brain volume. Accordingly, a patient with + 5 brain-PAD years has 15 % added risk of a future dementia diagnosis compared to one with 0 brain-PAD years, whilst keeping the covariates equal (e.g., both are 65-year-old females with similar MMSE scores and normalised brain volumes). Moreover, sensitivity analysis I, revealed that this risk increased to 6 % when the interval between neuroimaging assessment and dementia diagnosis was increased from a minimum of 3 months to at least 3 years. This could indicate that neuroimaging biomarkers are more informative in people further away from the development of manifest clinical symptoms of dementia.

Our findings are consistent with previous studies that used the brain-age paradigm to investigate subsequent dementia (Gaser et al., 2013, Wang et al., 2019). For example, the Wang et al. (2019) study showed that every year of brain-PAD had a 9 % increased risk of incident dementia, even when assessment preceded diagnosis by 5 years. Also, the Gaser et al. (2013) study reported a 10 % increased risk in converting from mild cognitive impairment to Alzheimer’s disease with a 3-year follow-up. Some important differences exist between our study and these previous reports, particularly regarding sample characteristics. In Wang and colleagues’ study, the sample was a population-based cohort in which participants who developed dementia were compared to those who did not and hence the latter are likely to have included many healthy participants. Instead, all our controls were patients who were referred to a memory clinic and who did eventually get a diagnosis, often a psychiatric or neurological one. Hence, the case-control contrast in the Wang’s study is expected to be larger than ours given that patients with psychiatric and neurological disorders are reported to show larger brain-PADs than healthy participants (Cole and Franke, 2017, Kaufmann et al., 2019, Cole et al., 2019). In our study, brain-PAD remained significantly associated to future dementia despite our sample including co-morbidities and complex medical histories.

Another important finding came from sensitivity analysis II. In patients with normal cognitive functioning (MMSE ≥ 27), brain-PAD remained significantly linked to progression to a dementia diagnosis. It is precisely when routine assessments in the memory clinic yield ambiguous results that the clinician may consider other more invasive or expensive tests to aid decision-making. This further illustrates the potential role of quantitative neuroimaging biomarkers in the clinic for augmenting clinical decision-making. For example, it is common for memory clinic patients with poor cognition to have ‘age-appropriate atrophy’ reported by a radiologist on qualitative assessment of their MRI scan. Quantitative neuroimaging analysis may help reconcile such findings.

In this study, we did not seek to demonstrate that brain-PAD is superior to the other covariates (age, MMSE, normalised brain volume, sex) in the association with risk of future dementia, instead we aimed to examine whether brain-PAD is valuable in addition to these established factors. Chronological age had the highest hazard ratio of the predictors (see Table 2), but brain-PAD remained significant even when including age and other covariates in the model, supporting the idea of brain-PAD having *additional* value when modelling dementia risk. The intercorrelations and VIF values of the model variables did not indicate problematic collinearity. These statistics provide reassurance that our model interpretation that brain-PAD adds value to the risk estimation of future dementia, is sound.

The MRI scan may have been used for different purposes along the patient pathway. For example, diagnostic support, prognosis, and exclusion of structural lesions or differential diagnosis. However, it is important to note that in our clinical experience, a dementia diagnosis is primarily based on a clinical and cognitive assessment, not an MRI scan. Given that the aim of this study was to test whether an MRI scan is indicative of risk of a *future* diagnosis of dementia, we wanted to minimise inclusion of cases where the MRI scan was used to inform a diagnosis at the time of scan. To guard against this, first we excluded cases where a dementia diagnosis was made before time of MRI scan. Second, we set a threshold of at least 3 months after time of scan, such that patients diagnosed with dementia before this time were also excluded. This 3-month threshold is the expected timeframe to receive a diagnosis based on our local clinical guidelines. Thirdly, as an additional conservative measure we ran a sensitivity analysis with a 3-year threshold, and the hazard ratio for brain-PAD remained the same. In summary, MRI may be used to inform a diagnosis of dementia at different timepoints, however this study indicates that neuroimaging biomarkers like brain-age may provide additional prognostic information, even in highly heterogeneous clinical populations. Importantly, given the common use of T1-weighted volumetric MRI in memory clinics, the brain-age biomarker can be provided at no additional financial cost or inconvenience to the patient.

Using MRI to detect early risk of dementia has other clear advantages (Johnson et al., 2012). MRI can provide dementia-specific information on patterns of atrophy, unlike blood or CSF biomarkers. MRI is more widely available, cheaper and less invasive than PET and involves no radiation, unlike CT. Moreover, MRI-derived biomarkers are objective, quantitative and exempt from practice effects, unlike clinical and cognitive assessments. Nonetheless, MRI may not be accessible to all patients, particularly those with contraindications, claustrophobia or severe illness. Other disadvantages include high demand leading to limited availability, cost, and the limited diagnostic and prognostic precision in certain cases.

One major strength of this study is having used data from pre-existing standard memory clinic rather than by experimental, and hence, artificial design. This confers two main advantages. The first is added ecological validity. This means that our findings are more representative of our target population - memory clinic patients. Our sample is drawn from south-east London and the population is characterised by a relatively diverse ethnic background (see Appendix D). This suggests our findings are robust to ethnic differences. While south-east London is not fully representative of nationwide memory clinic patients (Perera et al., 2016), the ethnic and socio-economic diversity are at least equal to or greater than many other parts of the UK. Crucially, our dataset overcomes selection bias, a bigger driver of unrepresentativeness and poor generalisability. There was no selection per se, other than the clinical decision to refer the patient for neuroimaging assessment based potential dementia risk – exactly the target population for markers aimed at early dementia identification. Secondly, it illustrates the feasibility of implementing clinical models involving quantitative neuroimaging biomarkers, which could be automatically integrated with other clinical data (e.g., radiology reports or electronic health records). Connecting neuroimaging biomarkers like brain-age with electronic health records has broad potential; it could aid the memory clinician with prognosis and support clinical trials including stratification, staging of disease severity and outcome markers (Jack et al., 2018) and, neuropsychiatric research at large (Kaufmann et al., 2019, Franke and Gaser, 2019, Cole et al., 2019).

To contextualise our findings, we considered how the effect size of the risk conferred by brain-PAD compared to common dementia risk factors. The study by Gottesman et al. (2017) reports *HRs* (*CI*) for incident dementia of 1.14 (0.99–1.31), 1.39 (1.22–1.59) and 1.77 (1.53–2.04) respectively for obesity, hypertension and diabetes relative to normal healthy conditions. Comparing these to brain-PAD is not straightforward considering study differences such as sample characteristics and covariate adjustments. However, for purely illustrative purposes, one can convert these *HRs* to brain-PAD years based on our model, by subtracting 1 from both the disease *HR* and brain-PAD’s *HR* and then dividing the former by the latter (e.g., [1.14–1] / [1.03–1] = 4.7), this would give + 4.7, +11.8 and + 20.5 brain-PAD years, respectively. Broadly speaking, this would mean that someone with a brain looking 11.8 years older than their chronological age would have a similar long-term risk of incident dementia to someone classified as having hypertension. Memory clinic patients may present with complex medical histories. For example, they may display poor cognition at baseline, as reflected by low MMSE scores, due to conditions other than a dementia diagnosis, such as stroke, tumour or psychosis. In fact, such differential diagnosis is a key clinical challenge. Our model is agnostic to the patient’s medical history (aside from excluding those with dementia diagnosis at or before baseline). Embracing the real-world heterogeneity of memory clinic patients was a deliberate choice in our study design, which aimed to maximise the model’s clinical utility by minimizing the information required to calculate dementia risk.

Various limitations of this study should be acknowledged. The first pertains to false negatives and false positives. The *noDD* group was defined on a negative search result for a dementia diagnosis, however, it is possible that some developed dementia that was unrecorded. Nevertheless, if our sample did contain such false negatives then, the contrast between *futureDD* versus *noDD* would reduce and consequently, our current finding would reflect a more conservative version than its true value. False positives - mistaken cases of *futureDD* patients - are less likely though not impossible, despite constant efforts at improving validation of information extracted from electronic health records (Perera et al., 2016, NIHR BRC, 2021). Future efforts in this direction could include enhanced triangulation of observations by linking electronic health records to additional databases, such as general practitioner records. A second limitation pertains to the precision of the timestamps of electronic health records events given the lack of consistency concerning whether the date reflects the time of the clinical event or the time the clinical event was recorded on the system. However, we assume that any such inconsistency would occur randomly across groups and not by a large interval. Thirdly, brain-age was calculated using only one modality of structural MRI scans. Although a T1-weighted brain-age model has been extensively validated across many studies it leaves a rich resource of common clinical scans, T2-weighted ones, unused. A multimodal brain-age model could improve the utility of this biomarker (Cole, 2020). Another caveat is that we cannot rule out that using a different algorithm to predict age would alter the findings. However, any differences are unlikely to be substantial given that published work using a similar voxel-based PCA brain-age approach showed that different regression models did not greatly impact performance accuracy (Baecker et al., 2021). Moreover, the brainageR model used in this study has been tested and validated previously, with a track-record of high accuracy (Clausen et al., 2022). In addition, Wang and colleagues reported similar findings whereby brain-PAD related to subsequent dementia, using a convolutional neural network model (Wang et al., 2019). Finally, although strength of the brain-age paradigm is in the simplicity of the multivariate-to-univariate transformation (i.e., many voxels reduced to a single brain-age value), ongoing developments in brain-age modelling provide localised brain-age estimates (Popescu et al., 2021) that could allow for novel composite brain-age scores of brain regions known to be critical to dementia (e.g., temporal lobe versus whole brain).

## 5 Conclusions

Overall, our findings demonstrate the value of using brain-age as a sensitive biomarker that has the potential to be used early‐on in memory clinics to detect patients at higher risk of developing dementia. This ‘real-world’ study further paves the way for the role of quantitative neuroimaging in bridging the gap between basic research and clinical applications, in particular, prognostication of dementia syndromes.

## CRediT authorship contribution statement

**Francesca Biondo:** Conceptualization, Data curation, Project administration, Formal analysis, Investigation, Writing – original draft, Visualization. **Amelia Jewell:** Conceptualization, Data curation, Project administration, Resources. **Megan Pritchard:** Conceptualization, Data curation, Project administration, Resources. **Dag Aarsland:** Conceptualization, Writing – review & editing. **Claire J. Steves:** Conceptualization, Writing – review & editing. **Christoph Mueller:** Conceptualization, Writing – review & editing. **James H. Cole:** Conceptualization, Data curation, Formal analysis, Investigation, Supervision, Writing – original draft.

## Declaration of Competing Interest

JHC is a scientific advisor to and shareholder in Brain Key and Claritas HealthTech, both medical image analysis companies. The other authors declare that they have no known competing financial interests or personal relationships that could have appeared to influence the work reported in this paper.

## Figures and Tables

**Table A1:** MRI acquisition parameter types.

MRI 'type'	Count	voxel dimensions (mm) x*y*z			Repetition time	Echo Time	Matrix size x*y*z			Slice thickness	Slice gap	Flip Angle	FoV	Inversion Time
1	433	1.25	1.25	1.2	8.592	3.8	192	192	180	1.2	1.2	8	240	1000
2	221	1.25	1.25	1.2	8.976	3.8	192	192	166	1.2	1.2	8	240	600
3	173	1.25	1.25	1.2	9.184	3.9	192	192	166	1.2	1.2	8	240	600
4	148	1.25	1.5	1.2	8.536	3.772	192	160	180	1.2	1.2	8	240	1000
5	53	1.25	1.5	1.2	8.932	3.776	192	160	166	1.2	1.2	8	240	600
6	45	1.25	1.25	1.2	8.812	3.9	192	192	180	1.2	1.2	8	240	1000
7	37	1.25	1.5	1.2	9.14	3.876	192	160	166	1.2	1.2	8	240	600
8	15	1.25	1.5	1.2	8.752	3.872	192	160	180	1.2	1.2	8	240	1000
9	2	1.25	1.25	1.2	8.976	3.8	192	192	150	1.2	1.2	8	240	600
10	1	0.859375	1.14583333	1.5	12.308	5.312	256	192	124	1.5	1.5	20	220	450
11	1	0.859375	1.14583333	1.5	12.428	5.348	256	192	124	1.5	1.5	20	220	450
12	1	1.25	1.25	1.2	9.192	3.904	192	192	136	1.2	1.2	8	240	600
13	1	1.25	1.25	1.2	8.984	3.804	192	192	138	1.2	1.2	8	240	600
14	1	1.25	1.5	1.2	9.148	3.88	192	160	140	1.2	1.2	8	240	600
15	1	1.25	1.5	1.2	9.14	3.876	192	160	144	1.2	1.2	8	240	600
16	1	1.25	1.25	1.2	8.976	3.8	192	192	146	1.2	1.2	8	240	600
17	1	1.25	1.5	1.2	9.14	3.876	192	160	148	1.2	1.2	8	240	600
18	1	1.25	1.5	1.2	9.14	3.876	192	160	154	1.2	1.2	8	240	600
19	1	1.25	1.25	1.2	9.184	3.9	192	192	160	1.2	1.2	8	240	600
20	1	1.25	1.25	1.2	9.06	3.792	192	192	166	1.2	1.2	8	240	600
21	1	1.25	1.25	1.2	9.952	4.012	192	192	166	1.2	1.2	8	240	600
22	1	1.25	1.5	1.2	8.62	3.792	192	160	180	1.2	1.2	8	240	1000

**Appendix C:**
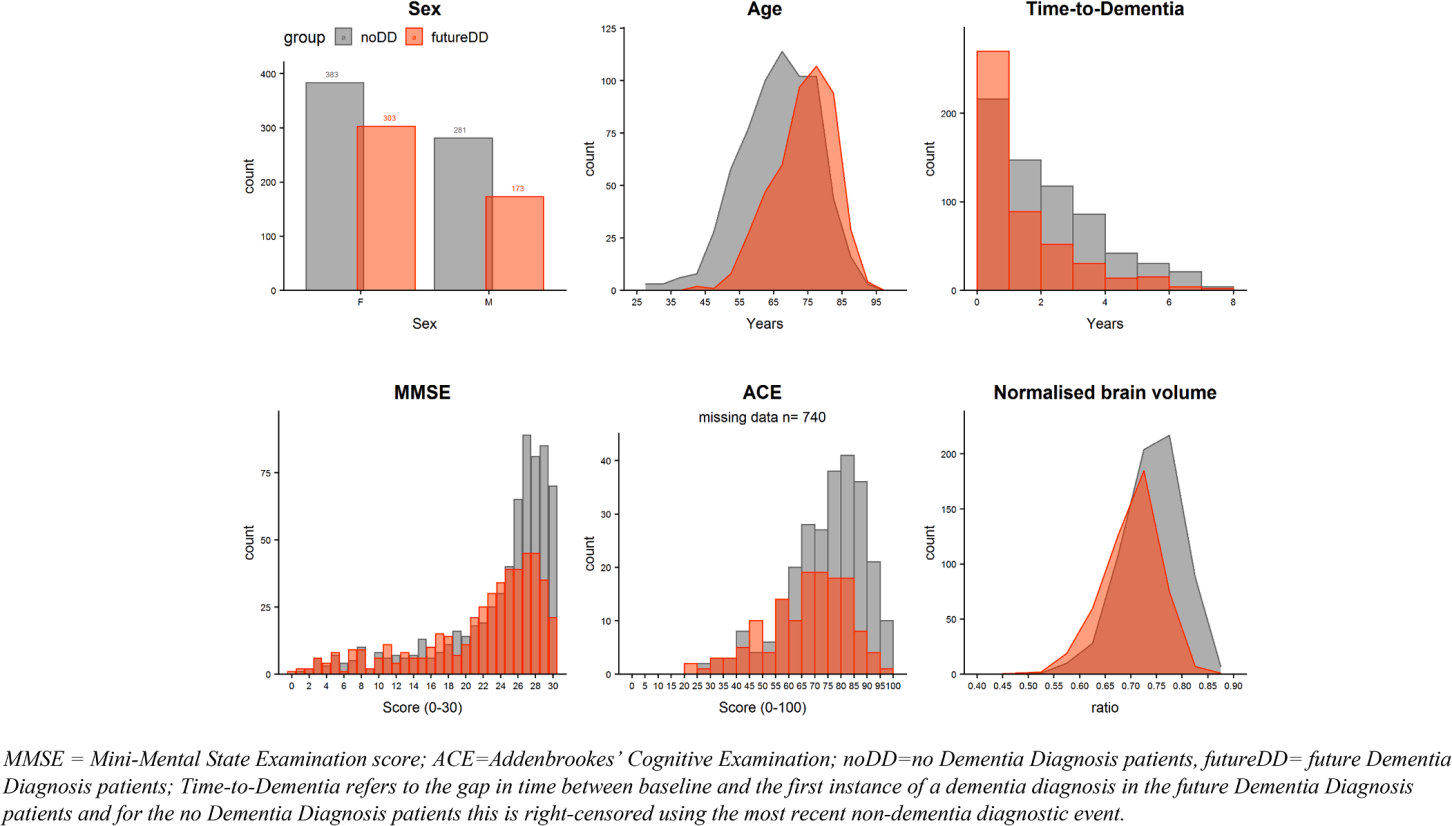


**Fig. D1:**
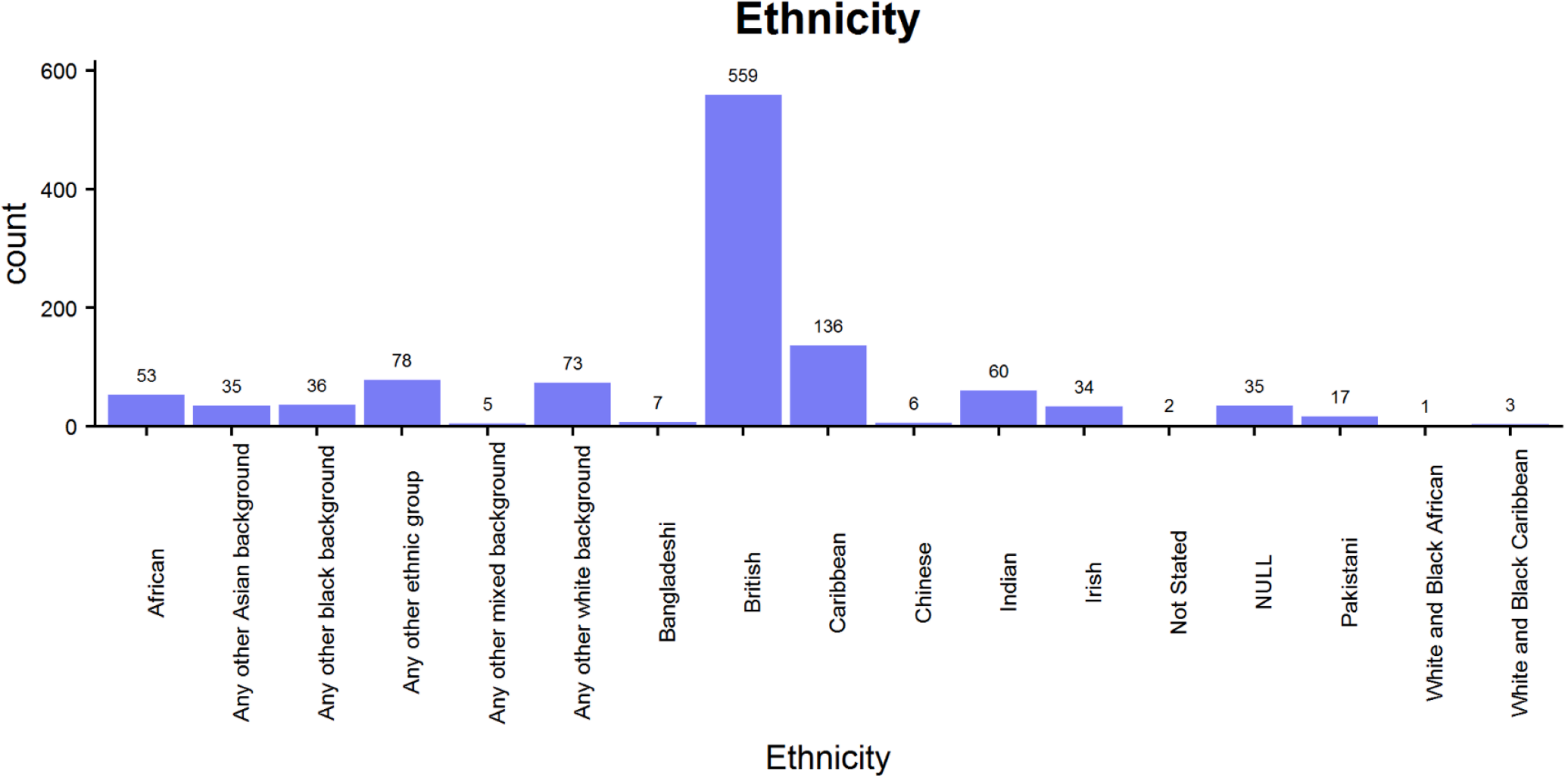
Ethnic background. Distribution of total sample split by 15 ethnic background groupings excluding two response categories for which there were no data available (“Not Stated” and “NULL”).

**Table E1:** Correlation matrix of model variables.

	age	MMSE	normalised brain volume	Time-to-Dementia
brain-PAD	−0.36	−0.01	0.06	−0.06
Age		−0.03	−0.45	−0.11
MMSE			0.11	0.09
normalised brain volume				0.09

Values indicate Pearson’s correlation coefficients.

**Table E2:** Variance Inflation Factors (VIF).

	**VIF**
**Main analysis**	
brain-PAD	1.24
age	1.54
age^2^	1.28
sex (M)	1.04
MMSE	1.02
normalised brain volume	1.12

## Data Availability

The authors do not have permission to share data.

## Appendix A

**MRI acquisition parameters.** All 1140 MRI scans were T1-weighted and obtained from the same GE 1.5 T MRI scanner (scanner A) at the Department of Neuroimaging, Institute of Psychiatry, Psychology and Neuroscience, King’s College London, UK. The MRI acquisition parameters varied slightly with a total of 22 types which are presented in Table A.1 including the count for each type.

## Appendix B

**BrainageR**. Detailed information on the brainageR model can be retrieved here (https://github.com/james-cole/brainageR). The brainageR model for v2.1 was trained on n = 3377 healthy individuals (mean age = 40.6 years, SD = 21.4, age range 18–92 years) from seven publicly-available datasets (see list below) and tested on n = 857 (mean age = 40.1 years, SD = 21.8, age range 18–90 years).

Training datasets:

• Australian Imaging, Biomarker & Lifestyle Flagship Study of Ageing (AIBL)
• Dallas Lifespan Brain Study (DLBS)
• Brain Genome Superstruct Project (GSP)
• IXI
• Nathan Kline Institute Rocklands Sample Enhanced
• Open Acces Series of Imaging Studies-1 (OASIS-1)
• Southwest University Adult Lifespan Dataset (SALD)

Testing dataset:

• camCAN

## Appendix C

Below is a pictorial version of baseline characteristics as presented in Table 1.

## Appendix D

### Appendix E

We assessed the variance inflation factor (VIF) of the predictors in our model (Table E.2). VIF indicates how much the standard error of a predictor variable would change due to the inclusion of a potentially collinear covariate. We note that the VIF is<2 for all predictors. Generally, VIF values of above 10 or 5 are thought to indicate problematic amounts of collinearity with others suggesting 2.5 as a more conservative threshold (James et al., 2013, Johnston et al., 2018).
